# Production of pastrami from turkey meat, and based on its physicochemical, microbiological, textural, and sensory characteristics using multivariate statistical analyses

**DOI:** 10.3389/fnut.2026.1943445

**Published:** 2026-09-18

**Authors:** Mustafa Çavuş, Hasan Cankurt, Tarık Ertürk, Kemal Durusu

**Affiliations:** 1 Food Technology Program, Food Processing Department, Safiye Çıkrıkçıoğlu Vocational School, Kayseri University, Kayseri, Türkiye; 2 Food Engineering Department, Faculty of Chemistry and Metallurgy, Yıldız Technical University, Istanbul, Türkiye; 3 Culinary Arts Program, Hotel, Restaurant and Catering Services Department, Develi Hüseyin Şahin Vocational School, Kayseri University, Kayseri, Türkiye

**Keywords:** dry curing, pastrami, PCA, texture, turkey meat

## Abstract

**Background:**

Turkey meat is an important source of animal protein for its nutritional value.

**Methods:**

In this study, principal component analysis (PCA) and hierarchical clustering analysis (HCA)were applied to examine the physicochemical, microbiological, sensory, and textural properties of turkey pastrami and pastrami samples produced from turkey breast meat purchased at the Kayseri market.

**Results:**

As a result of the analysis, the moisture content of the pastrami samples was determined as 45.25–48.95%, pH value as 5.88–5.96, salt content as 7.43–9.62%, ash content as 8.09–12.24%, water activity (aw) as 0.78–0.85, protein value as 34.62–38.80%, fat value as 2.03–17.91%, fenugreek thickness as 2–4 mm, L* value as 27.80–44.31, a* value as 7.48–18.47, and b* value as 6.89–12.25. In the microbiological analysis results, total mesophilic aerobic bacteria (TMAB) were determined to be 3.48–6.62 (log cfu/g) only in antrikot pastrami (AP) obtained from Kayseri market samples, coliform count was 2.44 (log cfu/g), yeast–mold count was 4.41 (log cfu/g) only in sliced pastrami (SP) samples obtained from Kayseri market, and enterobacteria count was 3.85 (log cfu/g) only in AP samples. Among the textural values, the best chewiness value was found in turkey pastrami (TP) samples produced from turkey breast meat. According to the sensory analysis results, TP received high scores from the panelists.

**Conclusion:**

It was determined that the moisture content of the pastrami samples used in the study was below the legal limit, the pH values were suitable for pastrami, the salt content was within the legal limits, and the highest protein value and best chewiness were found in TP samples. According to the principal component analysis and hierarchical clustering analysis, it was determined that the characteristics defining turkey pastrami are primarily the texture profile analysis parameters.

## 1 Introduction

The intake of foods necessary for people to grow, develop, be productive, and live long, healthy lives is called nutrition. Human beings need products of plant and animal origin for a healthy diet (1).

People can consume meat through various cooking techniques, as well as raw meat processed into products such as pastrami, sausage, and smoked meat. Meat products, besides increasing the shelf life of this product with high protein and nutritional value, enable the production of new products with different qualities. However, due to the high cost of red meat production, alternative sources are being investigated. For these reasons, the importance of poultry production and poultry meat is increasing day by day (2).

According to the legislation, pastrami refers to a cured and dried meat product obtained by properly separating pieces of meat from the carcasses of large animals, subjecting them to curing and washing processes in accordance with the technology, then pressing and drying processes, adding fenugreek, and drying again; it is a product that has not undergone heat treatment (3).

Pastrami is an important traditional meat product in Turkish culinary culture, characterized by its unique aroma, flavor, and appearance. Traditionally produced from healthy beef, pastrami is made through processes such as curing, brining, drying, equalization, and seasoning, and is not subjected to any smoking or heat treatment during production. This production method helps preserve the product’s unique sensory characteristics. In addition to preserving its nutritional value, pastrami has an extended shelf life and is one of the most widely consumed traditional meat products in Türkiye, which is considered an important component of cultural heritage (4).

Turkey meat is an important source of animal protein for its nutritional value. Turkey breeds with high live weight are raised worldwide. Due to the low live weights of domestic breeds in Türkiye, the use of hybrids with higher live weights and carcass yields has replaced them (5).

Turkey meat has a nutritional profile and flavor similar to those of chicken, although it generally contains less fat and cholesterol. Turkey breast, characterized by its high protein content and low connective tissue, is regarded as a high-quality source of animal protein (6). On average, 100 g of turkey breast provides 116 kcal of energy, 21.27 g of protein, 3.46 g of fat, and 74.23 g of moisture, with no detectable carbohydrates. It also supplies essential minerals, including potassium (306 mg), phosphorus (216 mg), sodium (37 mg), magnesium (24 mg), calcium (4 mg), iron (0.30 mg), zinc (1.01 mg), and selenium (12.3 μg), as well as vitamins A, B1, B2, B3, B6, and B12, contributing to its high nutritional value (7).

Turkeys (*Meleagris* spp.) account for approximately 5% of global poultry meat production (8). The global turkey stock was reported as 243,527,000 in 2024 (9). In Türkiye, the turkey stock was 2,827,000 in 2024 and increased to 3,192,000 in 2025. Turkeys accounted for approximately 0.8% of the country’s total poultry stock (10).

In turkey production, the carcass yield is approximately 74–75% of live weight, whereas this rate is 60–65% in cattle (11). Türkiye possesses many traditional meat products with unique aromas, flavors, and appearances. Pastrami, one of them, is produced from inspected beef.

This study aimed to produce pastrami with high protein and low-fat content by using turkey breast meat. The supplied turkey breast meat was processed in accordance with the technique, and turkey pastrami (TP) was obtained after about a one-month production process. In this way, turkey meat offers a solution to the search for alternatives in the meat industry and serves as an alternative raw material source to bovine carcass meat used in the pastrami sector in Turkey. The obtained TP is preferred by producers for its low cost and by consumers for its low cholesterol. Another aim of this study is to increase consumption of this meat by developing various meat products from a healthy protein source, such as turkey meat, and to spread consumption throughout the year. In this way, consumers will have access to both a high-nutrition meat product and an economical one.

In this study, we produced turkey pastrami using traditional processing methods. Furthermore, the physicochemical, textural, microbiological, and sensory characteristics of commercially available antrikot pastrami (AP) and sliced pastrami (SP) were comparatively evaluated using multivariate statistical analyses. The results provided a comprehensive assessment of the quality attributes of both traditionally produced turkey pastrami and commercial pastrami varieties, and the findings were interpreted accordingly.

## 2 Materials and methods

### 2.1 Materials

In this study, turkey breast meat was used to produce TP. Turkey meat was obtained from Kayseri market (Haşmet Tavuk). Turkey breast meat of suitable size was used for pastrami production. Rock salt to be used in the curing process and spices to be used in fenugreek were obtained from (Koçak Baharat). The salt to be used in the salting process was preferred to be 2–3-mm-sized rock salt. Fenugreek and garlic were also purchased from Kayseri market (Koçak Baharat). Production was carried out at Kayseri University Food Studies Application and Research Center. The antrikot pastrami (AP) and sliced pastrami (SP) samples compared in the study were obtained from Kayseri market.

### 2.2 Methods

#### 2.2.1 Preparation of pastrami samples

The production process for TP samples is visually documented in Supplementary Figure S1(a–l), and the sequential stages are presented as a flow chart in Figure 1. Furthermore, Figures 2a–d visually demonstrates the laboratory analyses and sensory evaluation procedures.

**Figure 1:**
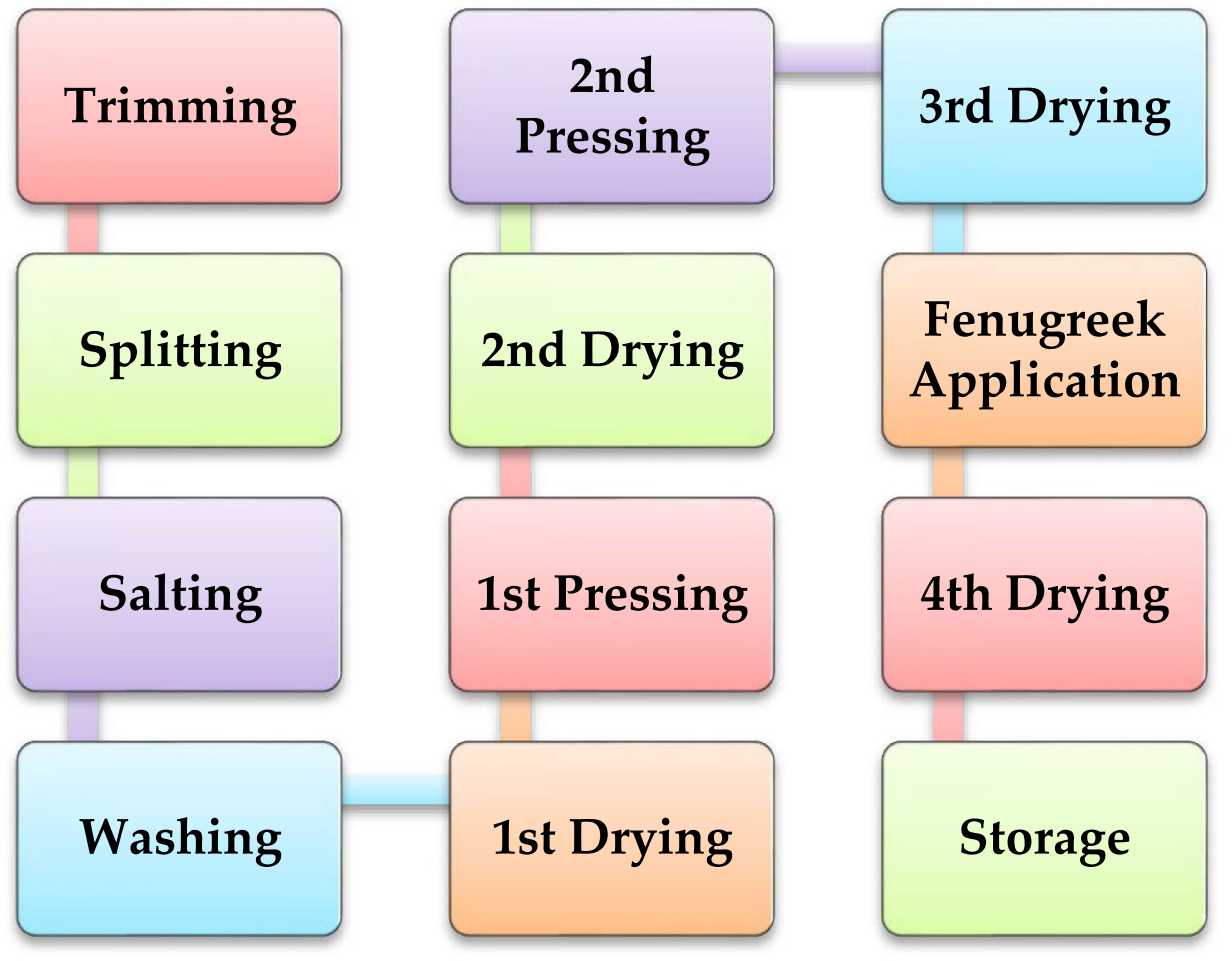
Flow chart of TP production.

**Figure 2:**
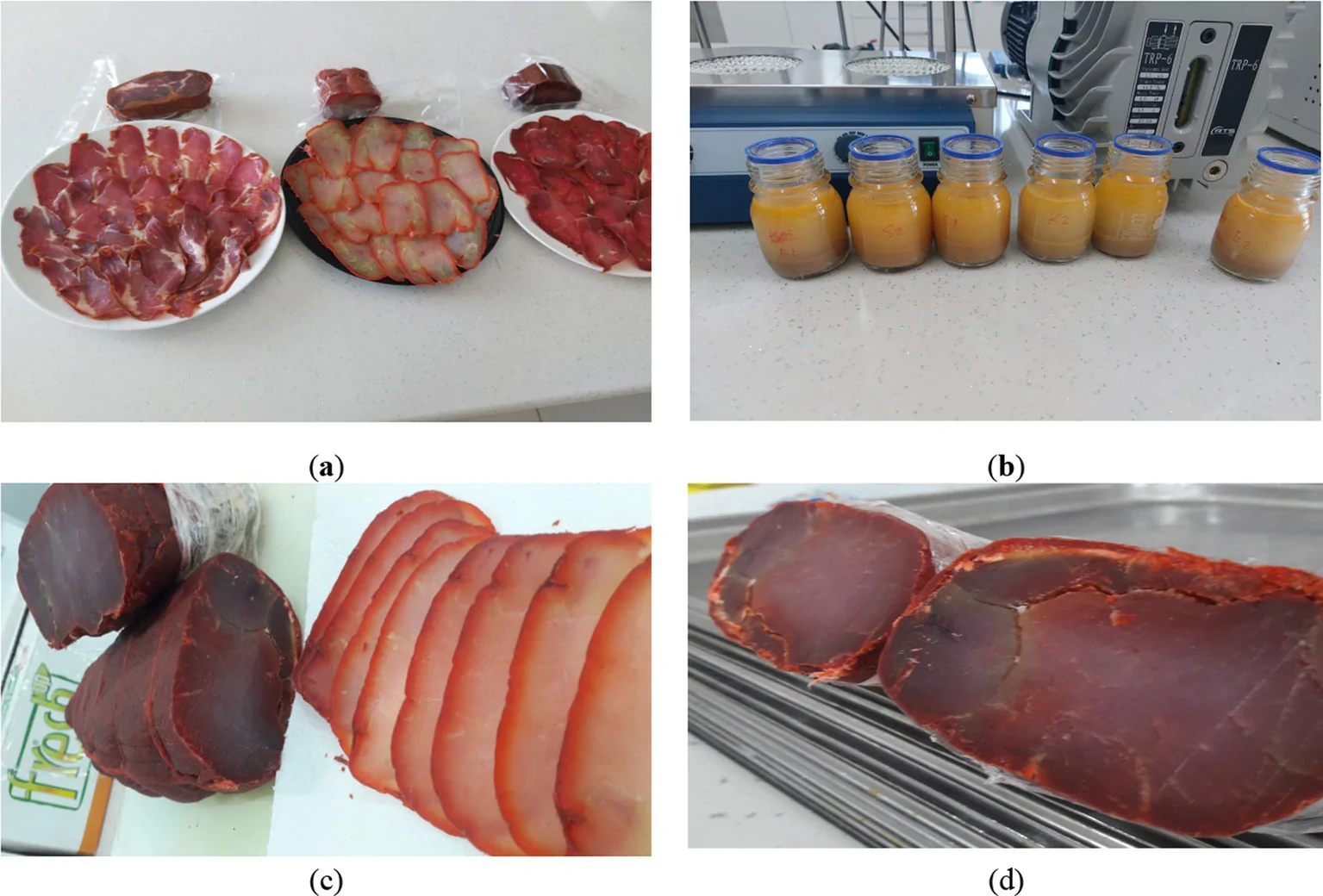
Images regarding the production process and laboratory analysis of TP samples **(a–d)**.

#### 2.2.2 Physicochemical analyses

For moisture analysis, the drying containers were placed in an oven (Nüve FN 120, Türkiye) for a few hours and weighed. Then, about 5 g of sample was weighed into these containers and dried at 105 °C for 24 h (12). For pH analysis of pastrami samples, 10 g of pastrami was weighed, 100 mL of pure water was added, and the samples were homogenized for about 1 min using a homogenizer (Wise Tis HG-15D). The probe of a pre-calibrated pH meter (Hanna, USA) was immersed in the samples, and measurements were recorded (13).

The salt content of the TP samples was analyzed according to the modified Mohr method (14). The ash content of the pastrami samples was analyzed in a muffle furnace (Elektromag, Turkey). For ash analysis, porcelain crucibles were first dried in an oven at 105 °C. Then they were taken to the desiccator, weighed, and tared. Approximately 3 g of sample was weighed into these crucibles. Then, after the pre-incineration process, the incineration process was carried out at 550 °C in the muffle furnace. After the incineration process, the crucibles removed from the muffle furnace were taken to the desiccator, allowed to cool, and weighed. The ash percentages of the pastrami samples were calculated (13).

To determine the water activity of pastrami samples, approximately 1 g of each sample was weighed. Then, the water activity values of the pastrami samples were determined using a pre-calibrated water activity device (Novasina, LabSwift-aw) (15).

Protein analysis of pastrami samples was carried out using the Kjeldahl method. Pastrami samples were homogenized, and fat content was determined by the Soxhlet extraction method. Approximately 10 g of pastrami sample was placed in the extractor and extracted for 5–6 h. After the extraction process, the samples were kept in the oven for about 30 min to remove any remaining solvents in the flask, and the sample weights were calculated (16). It was stated that the acceptable value should be between 1 and 4 mm (15).

#### 2.2.3 Color analyses

A reflectance colorimeter (HunterLab, Reston, VA, USA) was used to measure the color values of pastrami samples. L*, a*, and b* color values were determined by these measurements (17).

#### 2.2.4 Microbiological analyses

Total mesophilic aerobic bacteria (TMAB) enumeration in pastrami samples was performed on a principal component analysis (PCA) (Merck, Germany) using the smear method. The cultured media were kept at 30 ± 2 °C for 72 h and counted using a colony counting device, and the results were interpreted (18).

Violet Red Bile Agar (VRBA, Oxoid) was used to enumerate coliform-group bacteria in pastrami samples. The media were incubated at 30 °C for 2 days. Colonies formed at the end of incubation were counted (19).

Yeast–mold counts in pastrami samples were carried out using the smear method on Dichloran-Rose Bengal Chloramphenicol (DRBC) Agar (Merck, Germany) medium. The cultured media were incubated at 25 °C for 3–5 days. At the end of the period, these media were counted with a colony counter, and the counts were recorded (20).

Violet Red Bile Dextrose (VBRD) Agar (catalog number 1.10275, Merck Millipore, Germany) medium was used for the enumeration of Enterobacteriaceae in pastrami samples by smear method. The cultured media were incubated at 30 °C for 2 days, and colonies larger than 1 mm were counted (20).

#### 2.2.5 Sensory analyses

A hedonic scale was used to evaluate the sensory properties of pastrami samples produced with turkey meat and of AP and SP pastrami samples obtained from the Kayseri market. The samples were evaluated for appearance, color, odor, flavor, brittleness, and general acceptability by a pre-trained test team of 20 people aged between 20 and 60 years. The hedonic scale ranged from 9 to 1, with the lowest score of 1 indicating disliked features of the product (21).

#### 2.2.6 Textural analyses

For texture profile analysis, 1-cm-thick and 3.5 cm^2^ pastrami samples were analyzed using a TAXT Plus texture analyzer (Microstable, USA) with a P50 cylindrical probe (50-mm diameter). Test conditions were 1 mm/s speed, 35% penetration, 1 s wait time, 5.0 g trigger force, and 10 s application time (22).

#### 2.2.7 Statistical analyses

The physicochemical, microbiological, textural, and sensory properties of the pastrami in this study were evaluated by one-way analysis of variance (one-way ANOVA) in JMP PRO software. The differences between the means were tested using the Tukey multiple comparison test at the *p* < 0.05 level (23).

Principal component analysis (PCA), hierarchical clustering analysis (HCA), and heat map analysis were performed using XLSTAT software (XLSTAT, 2008, Addinsoft, New York, NY, USA) to categorize pastrami samples according to their physicochemical, microbiological, sensory, and textural properties. Additionally, correlations among all properties were determined using the Pearson correlation method using XLSTAT software.

## 3 Results

### 3.1 Physicochemical properties

The average moisture contents of pastrami samples were determined to be 45.25, 45.83, and 48.95% in SP, TP, and AP samples, respectively (Table 1). It was determined that the moisture values of the pastrami samples made with turkey meat were close to those of pastrami sold in the market. The TS 1071 Pastrami Standard states that the moisture content of pastrami should be at most 50%. The meat used in pastrami production, the salting method, the amount of salt used, the balancing process, the curing in fenugreek, the final drying process with fenugreek, and the storage conditions of the products directly affect the moisture content. A previous study (24) reported that the moisture values in pastrami samples from different beef meats were between 51.01 and 58.04%, and exceeded the notification value, and that slicing the samples and vacuum packaging caused the products’ moisture values to be high. Another previous study (25) determined moisture values in pastrami samples to be 36.39–45.37% and stated that the use of red pepper during the curing stage reduces water loss in pastrami.

**Table 1:** Physicochemical analysis results of pastrami samples.

Physicochemical analysis	Product name		
	AP	TP	SP
Moisture content (%)	48.95 ± 0.22^a^	45.83 ± 0.17^b^	45.25 ± 0.48^b^
pH	5.96 ± 0.01^a^	5.88 ± 0.01^b^	5.92 ± 0.01^ab^
Salt (%)	7.43 ± 0.35^b^	9.62 ± 0.71^a^	7.46 ± 0.14^b^
Ash (%)	8.09 ± 0.79^b^	12.22 ± 0.03^a^	8.44 ± 0.17^b^
Water activity	0.84 ± 0.00^a^	0.76 ± 0.01^b^	0.84 ± 0.02^a^
Protein (%)	37.94 ± 0.02 ^b^	38.80 ± 0.08 ^a^	34.62 ± 0.01^c^
Fat (%)	17.91 ± 0.31 ^a^	4.40 ± 0.07 ^b^	2.03 ± 0.03 ^c^
L*	32.69 ± 0.03^b^	44.31 ± 0.02^a^	27.80 ± 0.10^c^
a*	18.47 ± 0.02^a^	7.48 ± 0.01^c^	13.71 ± 0.13^b^
b*	10.43 ± 0.05^b^	12.25 ± 0.03^a^	6.89 ± 0.06^c^

^a–c^Lowercase letters indicate significant differences among pastrami samples (mean ± standard deviation (SD)) (*p* < 0.05). AP, Antrikot pastrami obtained from Kayseri market, SP, Sliced pastrami obtained from Kayseri market, TP, Pastrami produced from turkey breast meat.

When our study was analyzed, we found that the moisture content of pastrami made with turkey meat was lower than that of AP samples but very close to that of SP samples. It is thought that this difference between the pastrami samples is due to variations in production and overall meat composition. All pastrami samples used in the research conform to TS 1071 Pastrami Standard in terms of moisture content.

The pH of the meats used in pastrami production is very important. It has been determined that when high-pH meats are used in production, it is difficult to remove water from the meat, resulting in high water activity (a_w_) values. In addition, it has been determined that if such meats are used in production, the curing mixture diffuses more slowly into the meat, thereby prolonging the production process (26). The pH values were 5.88 for pastrami produced with turkey meat, 5.92 in SPs obtained from Kayseri market, and 5.96 for APs. It was determined that the pH values of pastrami samples made with turkey meat were close to those of pastrami sold in the market (Table 1). According to the Turkish Food Codex Communiqué on Meat and Meat Products, the pH value of pastrami should not exceed 6.00 (27). When our study results are examined, they are consistent with the communiqué. These findings are also similar to other studies. The variability of pH values in pastrami can be attributed to the drying process during production, proteolytic breakdown, the type and quality of the meat used, and finally differences in fermentation conditions. Sayın (25) determined the pH to be 5.69–5.95. İhtiyar (28), in his research on the quality of Kastamonu pastrami, reported that the pH of pastrami samples ranged from 5.74–6.52.

Salt values were determined to be 9.62% in pastrami produced with turkey meat, 7.46% in SPs obtained from Kayseri market, and 7.43% in APs (Table 1). It was determined that the salt content of pastrami samples made with turkey meat was higher than that of pastrami sold in the market. This is likely because turkey breast meat used in TP production contains 74.23% water. The water content of other meats used in pastrami production is around 72% on average. According to the TS 1071 Pastrami Standard, the salt content of pastrami should be a maximum of 8.5% of dry matter, though recent regulations have reduced this limit (29). While the pastrami samples obtained from Kayseri market and analyzedanalyzed conform to this standard, the TP produced contains non-standard salt. Adding salt to the pastrami by injection and extending the washing time will reduce the amount of salt.

In a study on pastrami, its salt content was determined to be 6.80% (30). The salt content of pastrami samples made from fish meat was determined to be 7.00% (31). In the context of other cured meats, average salt content can reach 7.13%, and specifically for pastrami sold in Konya, values around 6.15% have been observed (30). İhtiyar (28) determined the salt content in pastrami samples to be between 3.31 and 7.74%. Our results are consistent with those found in the literature.

Ash content values were determined to be 12.24% in pastrami produced with turkey meat, 5.56% in SPs obtained from Kayseri market, and 8.09% in APs (Table 1). The meat used in pastrami production plays a major role in the final product’s ash content. During the curing process, the amount of ash varies with the salt absorbed by the muscle structure. In the study investigating the effect of salting at different rates (3, 6, and 9%) on the amount of ash in pastrami production, 4.49% ash content was determined in the products subjected to the 3% curing process, 7.76% in the products subjected to the 6% curing process, and 9.86% in the products cured at 9% (32). Kuş (24) determined the ash content of pastrami samples to range from 4.93 to 6.06%.

The research determined that pastrami made with turkey meat had a higher ash content than the AP and SP samples. The ash content of pastrami samples sold in the market ranges from 4 to 9.30%. The ash values of pastrami samples made with turkey meat were found to be higher than those of pastrami samples sold in the market. The reason for this is that turkey breast meat used in the production of TP allows more salt to penetrate the pastrami during curing than beef, which is believed to increase the amount of ash.

The water activity of pastrami produced from turkey meat was determined to be 0.78, and that of AP and SP samples obtained from the Kayseri market was 0.84; it was observed that TP samples had lower water activity (Table 1). This is likely due to the high salt content of the turkey breast meat used in TP production. İhtiyar (28) stated that the average water activity values of Kastamonu pastrami were found to be 0.922 ± 0.016 for back pastrami and 0.915 ± 0.007 for kuşgömü pastrami. Akköse et al. (33) reported that water activity values for pastrami samples made from buffalo meat ranged from 0.85 to 0.90. During the dry curing process used in the production of pastrami, the osmotic pressure created by the intake of salt into the cell allows water to escape by diffusion. During this event, a significant mass transfer occurs in the product (34). Thus, the high water activity decreases, and the resulting pastrami becomes more resistant to microbial spoilage. Aw values of pastrami samples generally ranged from 0.85 to 0.90 (35).

The protein content of TP was 38.80%. While the protein content in AP samples was 37.94%, it was the lowest in SP samples at 34.62% (Table 1). The protein content of beef pastrami was determined to be 30.53%, chicken pastrami 40.13%, and TP 39.57% (36). Ren et al. (37) determined the protein values of pastrami samples as 47.4% in beef pastrami and 38.8% in sheep pastrami, and the protein content of pastrami produced from the rump of goat meat was determined as 46.2%. The protein content of fish pastrami samples produced with carp fish was determined as 21.27% (31). Kastamonu pastrami protein values ranged between 34.91 ± 5.33% in back pastrami and 37.69 ± 5.16% in kuşgömü pastrami. The fact that turkey breast meat contains more protein than the meat used in other pastrami samples led to the conclusion that the protein value of TP is high. Previous research supports our study.

In our study, the pastrami sample with the lowest fat content was an SP sample at 2.03%. It was determined that the fat content was 4.40% in TP samples and 17.91% in AP samples (Table 1). The high fat content in AP samples is due to the use of fatty meat. The low fat content in SP and TP samples is expected. Çakıcı (38) measured the total fat content of Kuşgömü, şekerpare, bohça, and back pastrami samples at 8.69, 5.05, 6.42, and 8.80%, respectively. Yımaz and Geçgel (39) determined the total fat content of three pastrami samples to be 14.89, 14.27, and 15.51%, respectively.

As a result of this study, when total fat values are analyzed, there are similarities to some studies and differences from others. These differences can be attributed to the varying fat content of the meats used during production, the supply of meats from different animal breeds, and variations in the production stage. According to the TS 1071 Pastrami Standard, the fat content of pastrami should be at most 40%.

Fenugreek thicknesses of pastrami samples produced from turkey meat and of pastrami samples obtained from Kayseri market were determined by measuring at different surfaces. As a result of the research, the thickness of fenugreek was determined to be 4 mm in pastrami samples obtained from the Kayseri market. In the TP produced, the thickness of the fenugreek was determined to be 2 mm. According to the Turkish Food Codex communiqué on pastrami production, the thickness of fenugreek in pastrami should be between 1 and 4 mm. Fenugreek thicknesses of pastrami samples are in accordance with this communiqué.

### 3.2 Color properties

The L* value, an indicator of brightness, was 44.31 in pastrami produced with turkey meat, 36.69 for AP samples obtained from the Kayseri market, and 27.80 for SP samples. The a* value, which determines the red color intensity, was found to be 7.48, 18.47, and 13.71 in TP, AP, and SP, respectively. The b* value, another color parameter, was determined to be 12.25, 10.43, and 6.89 in turkey, AP, and SP samples (Table 1).

Color is among the important quality parameters of meat and meat products. Color may vary in meat and meat products. The reasons for this include muscle structure, lipid oxidation, oxygen consumption rate, myoglobin concentration, and structure (27, 40) reported specific L*, a*, and b* color values for meat samples. Another study examined the effects of curing agents and temperatures on the L*, a*, and b* color values of pastrami (41, 42) reported average color values (L*, a*, and b*) for Ankara Erkeç pastrami varieties in their study.

As a result of the research, it was determined that the pastrami produced with turkey meat was brighter and yellow in color compared to AP and SP, and the red color appearance was lower. The turkey breast meat used in TP production caused this. The low a* values are attributable to turkey breast meat having a lower myoglobin concentration than beef (43).

### 3.3 Microbiological properties

Total mesophilic aerobic bacteria counts (log cfu/g) in pastrami samples ranged between 3.48 and 6.62 log cfu/g, the highest being 6.62 ± 0.03 (log cfu/g) in AP pastrami samples. The highest was 6.40 ± 0.01 (log cfu/g) in SP samples, and the total mesophilic aerobic bacteria count was 3.48 ± 0.04 (log cfu/g) in TP samples. In the coliform bacteria analyses of pastrami samples produced from turkey meat and pastrami samples obtained from Kayseri market, 2.44 ± 0.06 (log cfu/g) was detected only in AP samples. It was not detected in TP and SP samples (Table 2).

**Table 2:** Microbial analysis results of pastrami samples (log cfu/g).

Microbial analysis	Product Name		
	AP	TP	SP
TMAB	6.62 ± 0.03^a^	3.48 ± 0.04^c^	6.40 ± 0.01^b^
Coliform	2.44 ± 0.06^a^	***	***
Total yeast–mold	***	***	4.41 ± 0.00^a^
Enterobacteriaceae	3.85 ± 0.05^a^	***	***

^a–c^Lowercase letters indicate significant differences among pastrami samples (mean ± SD) (*p* < 0.05). AP, Antrikot pastrami obtained from Kayseri market, SP, Sliced pastrami obtained from Kayseri market, TP, Pastrami produced from turkey breast meat, TMAB, total mesophilic aerobic bacteria.

***Not detected.

In yeast–mold count, no yeast–mold was detected in TP and AP samples. It was determined as 4.41 ± 0.00 (log cfu/g) in SP samples. As a result of Enterobacteriaceae analyses, Enterobacteriaceae bacteria were not detected in TP and SP samples, while 3.85 ± 0.05 (log cfu/g) was detected in AP samples (Table 2). Batman (44) reported that the numbers of coagulase-positive staphylococci and sulfite-reducing anaerobic bacteria in all pastrami samples were below 10 and complied with the limits specified in the Turkish Food Codex. Sayın (25) stated that the number of Enterobacteriaceae in pastrami samples was below the limit in all samples. In addition, previous studies (45–47) reported that Enterobacteriaceae were below the limit values. Sayın (25) reported that the yeast–mold count in pastrami samples produced by using different ratios of red pepper was 4.59–6.27 (log cfu/g). However, the higher microbial loads observed in market-acquired pastrami than in turkey pastrami produced under controlled conditions suggest that standardized processing and hygiene protocols significantly impact final product safety (48, 49).

Aksu and Kaya (50) reported that the yeast–mold count of pastrami samples collected from the Erzurum market varied between 2 and 5.76 (log cfu/g); Karabıyıklı (51) reported that the yeast–mold count of pastrami samples varied between 1 and 4.83 (log cfu/g). Kabil (52) reported that the number of yeast–molds in pastrami varieties produced with different salt contents ranged from 3.48 to 7.07 log cfu/g, and the number of microorganisms belonging to the Enterobacteriaceae family was within the limit values. Hazar (53) determined the number of Enterobacteriaceae in pastrami varieties below the detectable limit. Hazar et al. (41) reported that the number of yeast–molds in pastrami samples produced by using different curing agents and curing temperatures ranged between 4.45–4.87 log cfu/g, and the number of Enterobacteriaceae was < 2 log cfu/g.

When the pastrami samples we examined in our study are evaluated for microbial quality, they are within the limits specified in the Turkish Food Codex Meat Products Communiqué.

### 3.4 Sensory properties

As a result of the sensory analysis, the panelists selected AP pastrami as the best, and it is thought that the appearance and fat content of AP pastrami samples were effective in this. SP and TP samples received similar sensory evaluation scores. It was determined that pastrami samples produced with turkey meat would be more desirable when the color parameters are improved (Table 3; Figure 3). Specifically, incorporating natural pigments such as beetroot or paprika extract into the curing or coating stages may harmonize the visual appeal of turkey-based pastrami with consumer expectations for traditional cured beef products. Such applications may offset the characteristic color weakness of turkey meat, resulting from its low myoglobin content, thereby increasing the market value of the product (54). In addition, vacuum packaging methods applied during storage have been observed to be effective in suppressing lipid oxidation and thereby preserving color stability, and in keeping the microbial load under control (55, 56). One study has demonstrated the direct effect of the salting and drying stages on the color parameters and oxidative stability of pastrami (57). Conversely, it has been reported that the smoking process reduces the moisture content of pastrami, thereby stabilizing its shelf life and sensory attributes, and produces particularly marked differences on days 30 and 60 (58). Statistical analyses have shown that differences in sensory scores among samples (*p* < 0.05) are particularly pronounced for texture hardness and chewiness parameters (59). These differences are attributed to variations in the protein and fat content of pastrami samples, as well as to the effects of different methods used during the curing stage on textural properties (60).

**Table 3:** Sensory analysis results of pastrami samples.

Sensory analysis	Product name		
	AP	TP	SP
Appearance	8.43 ± 0.67^a^	7.30 ± 1.42^b^	8.10 ± 0.72^a^
Color	8.48 ± 0.60^a^	7.00 ± 1.21^b^	7.80 ± 0.95^a^
Odor	8.12 ± 0.79^a^	6.80 ± 1.06^b^	7.40 ± 1.47^ab^
Flavor	8.23 ± 0.84^a^	6.90 ± 1.37^b^	6.98 ± 1.24^b^
Brittleness	7.80 ± 0.89^a^	7.00 ± 1.41^a^	7.05 ± 1.15^a^
General acceptability	8.20 ± 0.83^a^	6.90 ± 0.98^b^	7.10 ± 1.17^b^

^a–c^Lowercase letters indicate significant differences among pastrami samples (mean ± SD) (*p* < 0.05). AP, Antrikot pastrami obtained from Kayseri market; SP, Sliced pastrami obtained from Kayseri market; TP, Pastrami produced from turkey breast meat.

**Figure 3:**
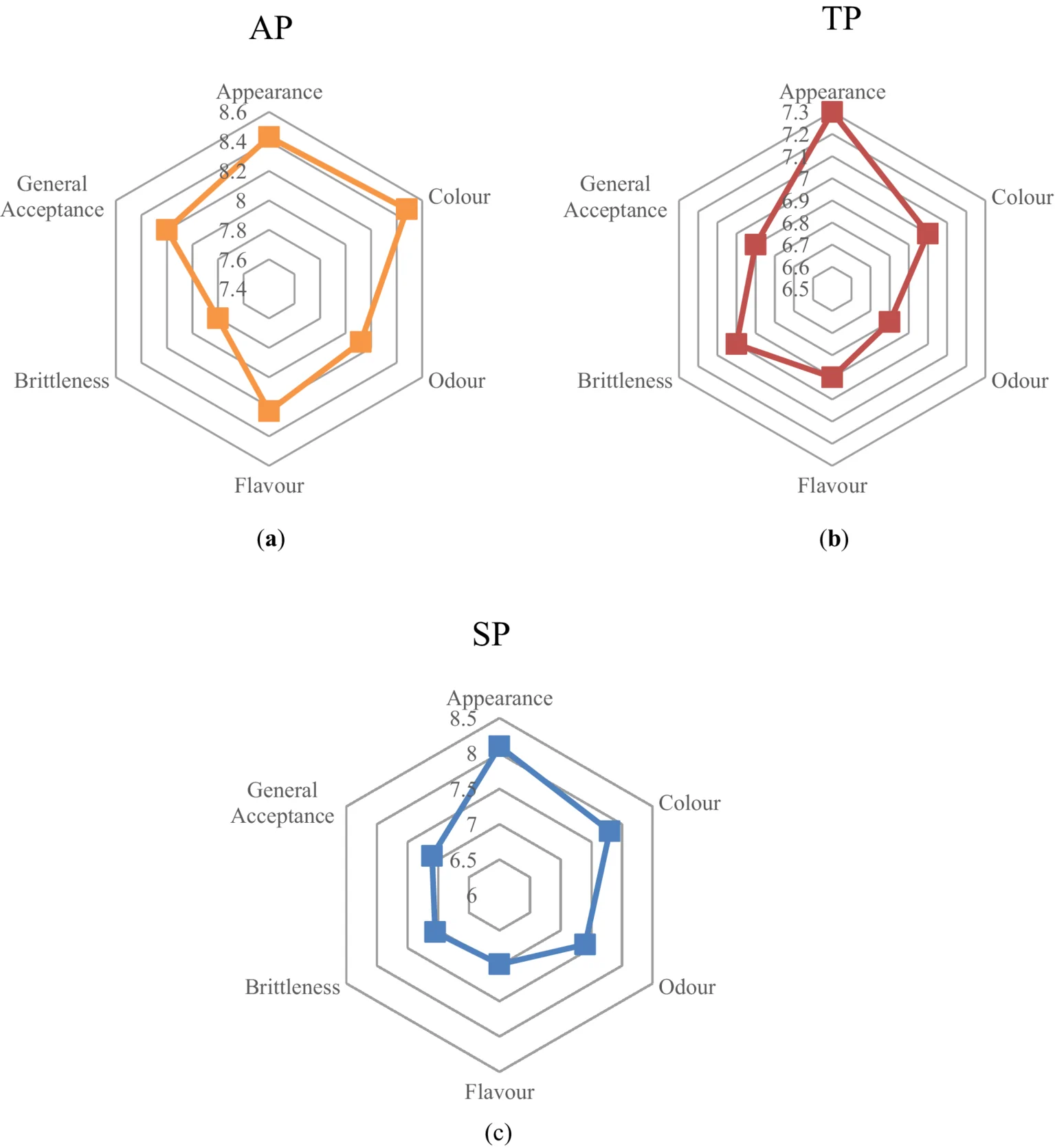
Sensorial properties of samples: **(a)** AP; **(b)** TP; **(c)** SP.

When the sensory analysis findings obtained from the present study were evaluated overall, AP pastrami samples received the highest scores in appearance (8.43 ± 0.67a), color (8.48 ± 0.60a), odor (8.12 ± 0.79a), flavor (8.23 ± 0.84a), and overall acceptability (8.20 ± 0.83a) parameters. In contrast, SP and TP samples were statistically at a similar level in overall acceptability (7.10 ± 1.17b and 6.90 ± 0.98b, respectively) and flavor (6.98 ± 1.24b and 6.90 ± 1.37b, respectively) parameters (*p* < 0.05). This indicates that the pastrami produced from turkey meat is appreciated by the panelists as much as the standard sliced pastrami on the market; however, the color (7.00 ± 1.21b) and appearance (7.30 ± 1.42b) scores of TP fall below the market threshold due to its low myoglobin content. The absence of a statistically significant difference in the crispiness parameter among the three pastrami varieties (all in the same letter group; *p* > 0.05) supports the view that turkey meat can serve as an alternative to beef meat from this perspective (21).

When our study’s findings are compared with Yetimoğulları’s Ankara Erkeç pastrami study, both studies show that muscle type significantly affects sensory acceptability (42). Yetimoğulları reported appearance scores in the 5.00–6.00 range and odor values in the 5.28 ± 0.40–6.06 ± 0.29 range; the most appreciated variety was kol pastrami. In our study as well, AP (8.43 ± 0.67) received the highest scores. In Batman’s infrared drying study, flavor was reported in the 2.00–3.30 range, color in the 2.20–3.40 range, appearance in the 2.10–2.90 range and texture in the 2.00–3.50 range; the infrared method was found superior in all parameters, and it was emphasised that pastrami produced from turkey meat would become more preferred by consumers if its color parameters were improved (44).

### 3.5 Textural properties

The hardness values of the analyzed pastrami samples ranged from 11,564 to 16,011 g. The hardness value was the lowest in AP samples and the highest in SP samples. The hardness value of TP was found between these two samples (14,534 g). The lowest adhesiveness value was found in TP samples (−5.923 g·s). The highest value was found in AP samples (−1.365 g·s). Springiness values of all pastrami samples were very close to each other (0.05–0.06). When cohesiveness values were analyzed, the lowest value was found in TP (0.727) and the highest value was found in AP samples (0.861). When gumminess values were analyzed, the highest value was found in SP samples (12,858), and the lowest value was found in AP samples (9,953). When chewiness values were analyzed, the highest value was found in pastrami produced with turkey meat (535.649). AP and SP (600.511–657.711) samples were found, respectively. In terms of resilience values, AP and SP samples were the same (0.42), whereas TP had a lower value (0.33) than these two samples (Table 4).

**Table 4:** Texture analysis results of pastrami samples.

Texture analysis	Unit	Product name		
		AP	TP	SP
Hardness	N	11,564.00 ± 1.42^c^	14,534.00 ± 2.83^b^	16,011.00 ± 1.42^a^
Adhesiveness	N·s	−1,365 ± 0.00^a^	−5,923 ± 0.00^c^	−2,322 ± 0.00^b^
Springiness	mm	0.06 ± 0.00^a^	0.05 ± 0.00^b^	0.05 ± 0.00^b^
Cohesiveness		0.86 ± 0.00^a^	0.73 ± 0.00^c^	0.80 ± 0.00^b^
Gumminess	N	9,953.00 ± 1.41^c^	10,569.00 ± 1.41^b^	12,858.00 ± 11.31^a^
Chewiness	N·m	600.51 ± 0.00^b^	535.65 ± 0.00^c^	657.71 ± 0.00^a^
Resilience		0.42 ± 0.00^a^	0.33 ± 0.00^c^	0.42 ± 0.00^b^

^a–c^Lowercase letters indicate significant differences among pastrami samples (mean ± SD) (*p* < 0.05). AP, Antrikot pastrami obtained from Kayseri market; SP, Sliced pastrami obtained from Kayseri market; TP, Pastrami produced from turkey breast meat.

In pastrami samples produced with buffalo meat, 36.89 N was found in bird pastrami, 25.56 N in back pastrami, 22.23 N in şekerpare pastrami, 15.31 N in bohça pastrami, and 12.51 N in shovel pastrami (33). Ekmekçi (61) stated that the chewiness values of pastrami samples were between 22.26–30.84 N. The chewiness value of a dry-cured meat product produced with lamb rump meat was found to be 3.54 N (62). Kuş (24) found that the hardness of pastrami samples averaged 21.05–26.32. Yetimoğulları (42) reported that the average hardness of Ankara Erkeç ranged from 36.73 ± 8.12 to 77.01 ± 11.65. Resilience values were found to be between 0.65 ± 0.08–0.78 ± 0.03; adhesiveness values were determined between 0.64 ± 0.05 and 0.75 ± 0.04; the average of the gumminess values were determined between 25.53 ± 5.04 and 57.71 ± 8.38; the average of the chewiness values was between 17.20 ± 3.67 and 45.28 ± 7.08; and the resilience value was between 0.27 ± 0.04–0.35 ± 0.03. The significant variations in these textural parameters among meat sources, particularly hardness and chewiness, underscore the need for standardized processing conditions to ensure consistent quality metrics (33). Furthermore, the observed textural differences among samples are likely attributable to distinct drying rates and moisture losses inherent to the specific muscle structures used in product manufacturing (64).

Batman (44) determined hardness values between 16.33 and 32.21 N, gumminess values between 64.10 and 84.84, chewiness values between 24.41 and 27.69, resilience values between 1.52 and 1.70 mm, and chewiness values between 25.99 and 30.52 in pastrami samples.

Akköse et al. reported that chewiness values in pastrami varieties produced from water buffalo meat ranged from 12.51 to 36.89 N, with the highest value determined in kuşgömü and the lowest in kürek pastrami varieties. Yetimoğulları reported that, in Ankara Erkeç pastrami varieties, hardness values ranged from 36.73 ± 8.12 to 77.01 ± 11.65 N, springiness from 0.65 ± 0.08 to 0.78 ± 0.03, cohesiveness from 0.64 ± 0.05 to 0.75 ± 0.04, gumminess from 25.53 ± 5.04 to 57.71 ± 8.38, chewiness from 17.20 ± 3.67 to 45.28 ± 7.08, and resilience from 0.27 ± 0.04 to 0.35 ± 0.03, and indicated that kol pastrami samples exhibited the highest values for most textural parameters (42). Batman, on the other hand, reported that, in pastrami samples produced using different drying methods, hardness (16.33–32.21 N), adhesiveness (64.10–84.84), gumminess (24.41–27.69), springiness (1.52–1.70 mm) and chewiness (25.99–30.52) values were obtained, and stated that the open-air drying method yielded the highest results in terms of chewiness and adhesiveness (44). Kırkyol and Akköse reported that hardness, cohesiveness, and chewiness values increased throughout pastrami production, and that Warner-Bratzler shear force increased at the end of the first drying stage and in the final product (65). Kara et al. determined the TPA-hardness value in commercial pastrami samples as 8,222.25, which was higher than that of sucuk (5,320.21) and salam (1,672.10) samples but lower than that of sausage samples (3,265.27) (66). Hastaoğlu and Vural reported that substituting 15% KCl for NaCl was acceptable in terms of sensory properties. In contrast, the hardness and chewiness values of samples with a 30% KCl substitution were scored low by the panelists (67).

### 3.6 Multivariate statistical analyses

Pearson correlation analysis, principal component analysis, and two-way clustering heatmaps were performed using XLSTAT software, and the results are presented in Supplementary Table S1; Tables 5, 6; Figures 4a–f, 5. The clustering structure created using the Ward coupling method and Euclidean distance was evaluated for both samples and variables.

**Table 5:** Results of PCA analysis using data of all properties of the pastrami samples.

F	Eigenvalue	Variability (%)	Cumulative (%)
F1	15.882	52.941	52.941
F2	9.536	31.788	84.730
F3	2.381	7.937	92.667
F4	1.016	3.388	96.055
F5	0.524	1.746	97.801
F6	0.301	1.002	98.803
F7	0.141	0.468	99.272
F8	0.098	0.326	99.598
F9	0.054	0.181	99.779
F10	0.043	0.145	99.924
F11	0.020	0.068	99.992
F12	0.002	0.008	100.000
F13	0.000	0.000	100.000
F14	0.000	0.000	100.000

**Table 6:** Principal component coefficients (loadings) and their contributions.

Components	First loading		Second loading		Third loading		Fourth loading	
	Contributions		Contributions		Contributions		Contributions	
	PC1	(%)	PC2	(%)	PC3	(%)	PC4	(%)
Chemical composition								
Dry matter	0.641	0.411	**0.746**	0.556	−0.149	0.022	0.014	0.000
pH	**−0.947**	0.897	−0.214	0.046	0.150	0.023	0.066	0.004
Salt	**0.935**	0.874	−0.321	0.103	−0.064	0.004	0.004	0.000
Ash	**0.952**	0.906	−0.257	0.066	−0.108	0.012	−0.009	0.000
Aw	**−0.939**	0.881	0.332	0.111	0.077	0.006	0.011	0.000
Fat	−0.651	0.424	**−0.744**	0.554	0.148	0.022	0.000	0.000
Protein	0.376	0.141	**−0.925**	0.855	0.040	0.002	−0.034	0.001
Fenugreek thickness	**−0.925**	0.856	0.330	0.109	0.079	0.006	0.040	0.002
Color values								
*L*	**0.806**	0.650	−0.589	0.347	−0.041	0.002	−0.034	0.001
*a*	**−0.986**	0.972	−0.104	0.011	0.127	0.016	0.025	0.001
*b*	0.504	0.254	**−0.862**	0.744	0.020	0.000	−0.029	0.001
TPA parameters								
Hardness	0.495	0.245	**0.858**	0.735	−0.138	0.019	0.003	0.000
Adhesiveness	**−0.984**	0.969	0.139	0.019	0.103	0.011	0.031	0.001
Springiness	**−0.744**	0.553	−0.639	0.408	0.157	0.025	−0.042	0.002
Cohesiveness	**−0.985**	0.970	−0.105	0.011	0.128	0.016	0.027	0.001
Gumminess	0.010	0.000	**0.995**	0.990	−0.093	0.009	0.019	0.000
Chewiness	−0.679	0.460	**0.733**	0.538	0.012	0.000	0.034	0.001
Resilience	**−0.939**	0.881	0.333	0.111	0.079	0.006	0.033	0.001
Microbiological parameters								
Coliform	**−0.748**	0.560	−0.646	0.417	0.145	0.021	0.011	0.000
Total bacteria	**−0.956**	0.915	0.278	0.077	0.086	0.007	0.020	0.000
Enterobacter	**−0.748**	0.560	−0.646	0.417	0.150	0.022	0.007	0.000
Yeast–mold	−0.190	0.036	**0.979**	0.958	−0.068	0.005	0.025	0.001
Sensory parameters								
Appearance	−0.518	0.269	−0.007	0.000	−0.439	0.192	**−0.662**	0.438
Color	**−0.627**	0.393	−0.115	0.013	−0.500	0.250	−0.529	0.280
Odor	−0.506	0.256	−0.161	0.026	**−0.563**	0.317	−0.058	0.003
Flavor	−0.447	0.200	−0.363	0.131	**−0.615**	0.378	0.272	0.074
Brittleness	−0.325	0.105	−0.265	0.070	**−0.724**	0.524	0.360	0.129
General acceptability	−0.508	0.258	−0.347	0.120	**−0.665**	0.442	0.271	0.073

For each parameter, the factor loadings across PC1–PC4 were compared, and the value with the highest magnitude, regardless of its sign, is shown in bold.

**Figure 4:**
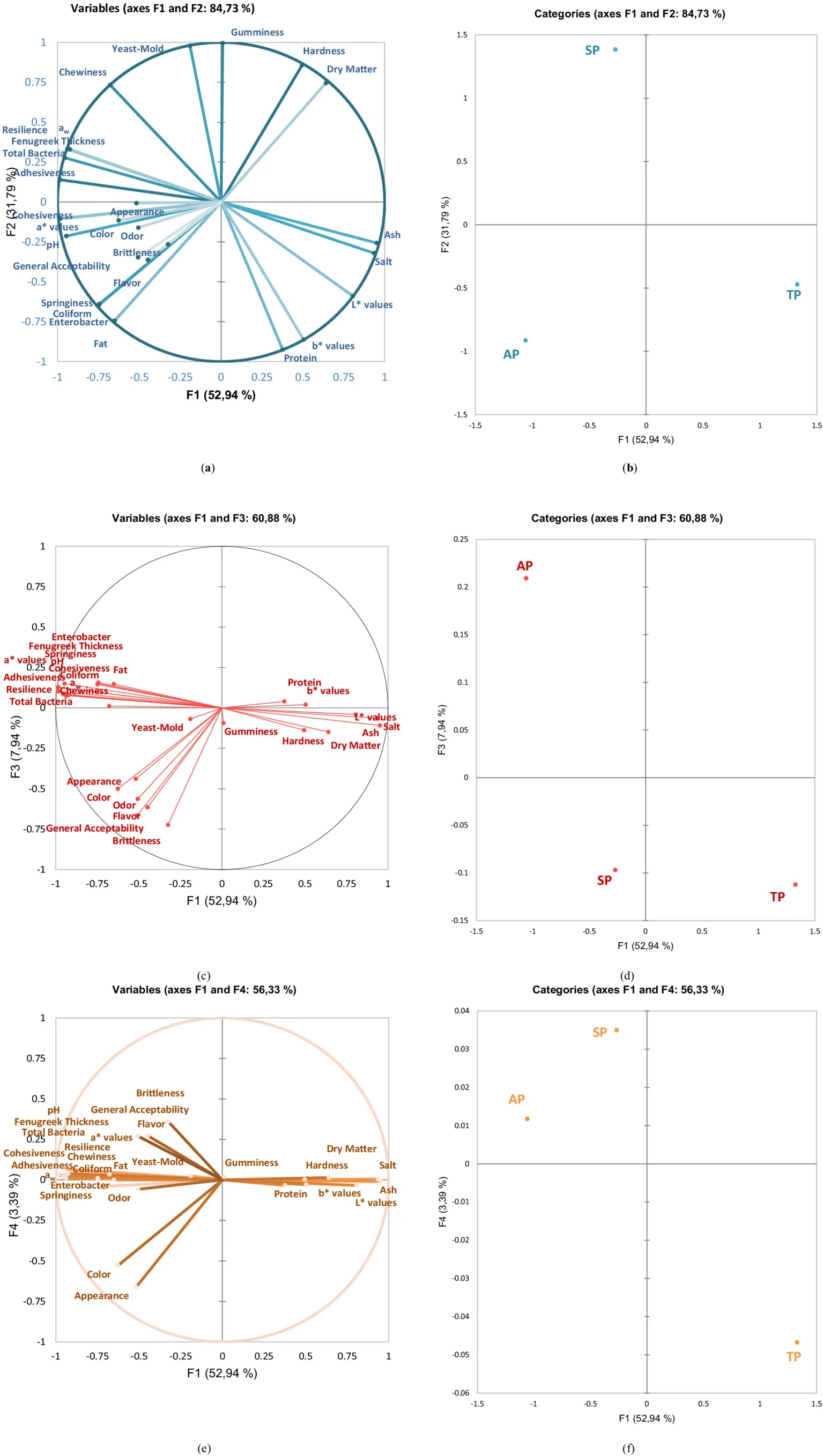
Plot of the PCs loading and score vectors: **(a)** Variables (axes F1 and F2), **(b)** Categories (axes F1 and F2), **(c)** Variables (axes F1 and F3), **(d)** Categories (axes F1 and F3), **(e)** Variables (axes F1 and F4), **(f)** Categories (axes F1 and F4).

**Figure 5:**
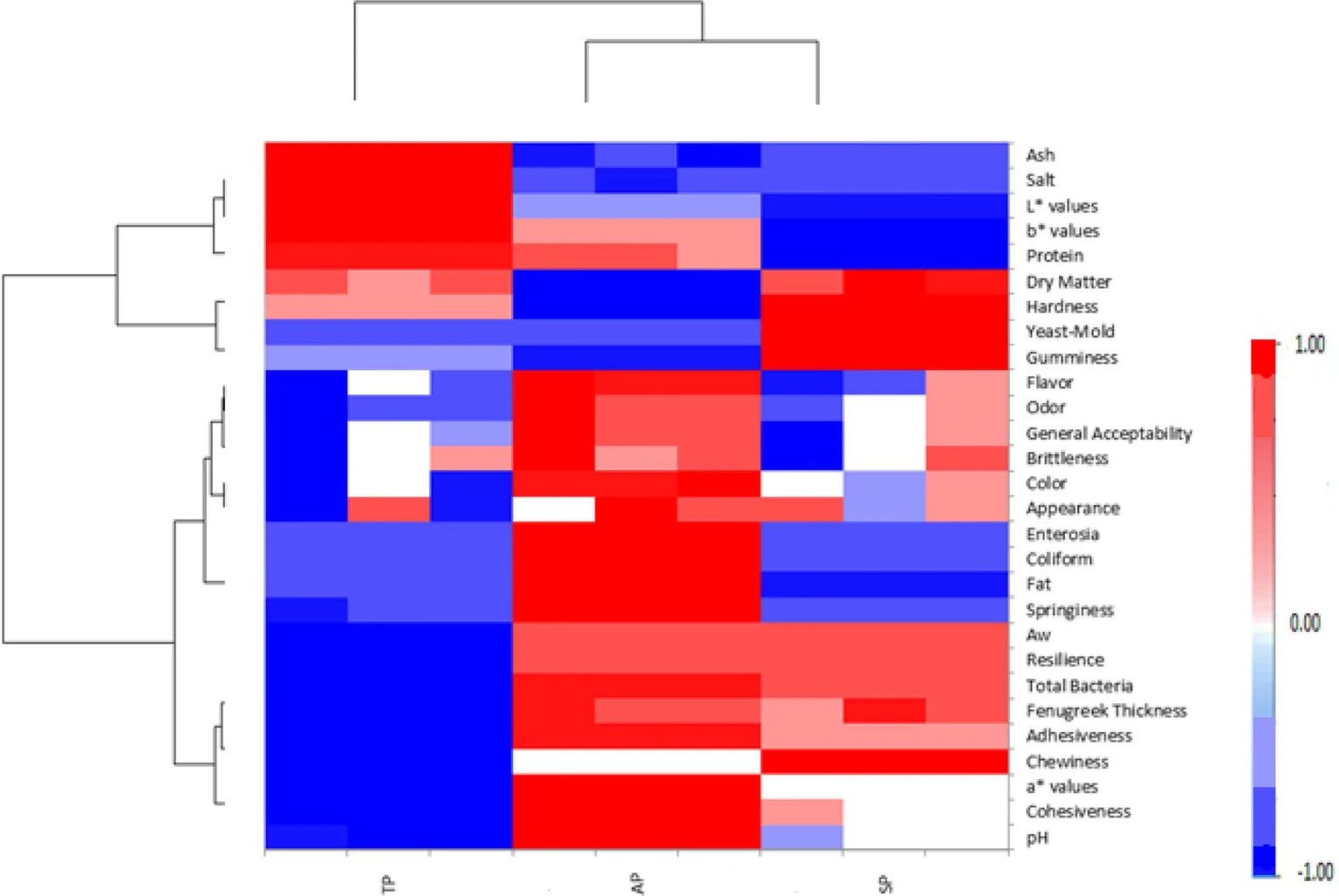
Heat map of physicochemical, textural, microbiological, and sensory compounds of pastrami analogs.

#### 3.6.1 Correlation results

The detailed correlation coefficients among all 28 variables are presented in Supplementary Table S1. There are several important correlation parameters between variables. For example, there are positive and negative correlations between hardness, gumminess, and springiness. The hardness value showed a positive correlation with gumminess and a negative correlation with springiness.

Regarding the correlations between TPA parameters, significant correlations were found. For example, adhesiveness showed a positive correlation with cohesiveness, resilience, and chewiness. Cohesiveness showed a positive correlation with springiness and resilience. In addition, chewiness showed a positive correlation with resilience. Gumminess showed a negative correlation with springiness.

Coliform showed a positive correlation with fat and a negative correlation with dry matter. Total bacteria showed a positive correlation with aw while a negative correlation with salt and ash. In addition, Enterobacteria showed a positive correlation with fat. Yeast–mold showed a negative correlation with protein and b* value.

pH showed a positive correlation with adhesiveness and cohesiveness, while showing a negative correlation with protein and b* value. In addition, protein showed a positive correlation with the b value and L value, while showing a negative correlation with gumminess, chewiness, and yeast–mold.

L* value showed a positive correlation with salt, ash, and *b* value, while showing a negative correlation with fenugreek thickness, adhesiveness, chewiness, resilience, total bacteria, and aw. a* value showed a positive correlation with adhesiveness, cohesiveness, resilience, total bacteria, pH, and aw, while a negative correlation with salt and ash.

Finally, appearance showed a positive correlation with color. General acceptability showed a positive correlation with brittleness and flavor.

#### 3.6.2 PCA results

In the literature, PCA and heat map analyses have been successfully used to correlate the physicochemical, textural, sensory, and microbiological properties of pastrami samples (Tables 5, 6; Figures 4a–f, 5) (68, 69). In this study, four principal components (PCs) were identified as significant in explaining the total variability of the product properties. Table 5 shows the eigenvalues and variances of each PC. As shown, the eigenvalues of PC1–PC4 were greater than 1. PC1, PC2, PC3, and PC4 explained 52.94, 31.78, 7.93, and 3.38% of the total variability in the dataset, respectively. In other words, 96.05% of the total variance across the 14 variables tested can be explained by four new variables (PCs). This percentage is sufficient to explain the variability, as it exceeds 70% (70). The PC coefficients (loadings) and contributions of the significant principal components are shown in Table 6. According to Table 6, the parameters explained the variation in the PCs as follows: results of PCA analysis using data on the physicochemical, microbiological, sensory, and textural properties of the pastrami samples.

Table 7 lists which principal components the variables belong to, is given below. Score graphs and loading PCs are shown in Figures 4a–f and F1-F2, F1-F3, and F1-F4 graphs.

**Table 7:** Variables belonging to the principal components.

Principal Components	Variables
PC1:	Adhesiveness, springiness, cohesiveness, resilience, coliform, total bacteria, Enterobacter, pH, Salt, Ash, Aw, L* values, a* values, fenugreek thickness, color
PC2:	Hardness, gumminess, chewiness, yeast–mold, protein, fat, b* values, dry matter
PC3:	Odor, flavor, brittleness, and general acceptability
PC4:	Appearance

From the F1-F2 score graph, it is seen that hardness is positively correlated with gumminess and dry matter content, as these are located in the same quartile. TP is located in the lower right quartile of the F1-F2 graph due to its high ash, salt, protein, L* and b* values. The clustering in the lower right quartile of the F1-F3 graph is due to positive correlations with protein and b* value. From the F1-F4 score graph, it is seen that L* value is positively correlated with protein, ash, and b* value, as these are located in the same TP quartile. The relationships observed from these score graphs have been explained in detail previously using the correlation analysis provided in Supplementary Table S1, along with Figures 4a–f.

#### 3.6.3 HCA results

In TP samples, ash, L* and b* values, salt, dry matter, and protein were high (red zone). In contrast, aw, resilience, total bacteria, fenugreek thickness, adhesiveness, chewiness, a value, cohesiveness, pH, yeast–mold, flavor, and odor values were low (blue zone).

In AP samples, flavor, odor, general acceptability, brittleness, color, appearance, enterobacter, coliform, fat, springiness, total bacteria, adhesiveness, a* value, cohesiveness, pH, and protein were high (red zone); ash, salt, L* value, dry matter, hardness, yeast–mold, and gumminess showed low (blue) values.

In SP samples, dry matter, hardness, yeast–mold, gumminess, and chewiness showed high values (red zone). In contrast, ash, salt, protein, fat, enterobacter, coliform, springiness, and L* and b* values were low (blue).

When the three products were compared, TP exhibited the most stable profile with high physicochemical and low textural loads, while AP exhibited high sensory acceptability and low physicochemical loads. SP exhibited high textural properties and low physicochemical properties. The inclusion of microbiological safety parameters as a separate group from textural and physicochemical parameters in the dendrogram of variables indicates that food safety and textural quality should be evaluated as independent dimensions in pastrami formulations. This multivariate approach validates the findings obtained with univariate analyses within an integrated framework.

## 4 Conclusion and recommendations

Water activity values in pastrami production are strongly influenced by the salt content of the product (29). While water activity is similar in pastrami samples produced from beef, it remains lower in pastrami produced from turkey. Water activity decreases rapidly with increasing salt content.

High salt content affects the ash content of pastrami samples. The high salt content of TP samples contributes to higher ash content. Based on protein analysis, the highest protein ratio was observed in TP samples. These results support our findings. In the sensory analysis, color is an important criterion for consumers’ liking (71). The meat product used in pastrami has a significant effect on color values. While pastrami samples produced from beef were red, pastrami samples produced from turkey meat were between red and pink.

When the textural analysis results were evaluated, the TP samples exhibited greater chewiness than the pastrami samples obtained from the Kayseri market. This result will improve the absorption and digestibility of pastrami produced with turkey meat.

Sensory quality parameters such as appearance, odor, flavor, brittleness, and general acceptability may differ significantly among pastrami samples (58). During the study, while consumers gave higher sensory scores to AP, TP’s preference level was the same as SP.

1. Pastrami produced from turkey breast meat exhibits panelist acceptability equivalent to that of the sliced pastrami available on the market; however, with its higher protein content (38.80%) and lower fat content (4.40%), it constitutes a healthy alternative (7). This finding further supports the conclusion that, from a sensory standpoint, turkey meat can be utilized as a raw material in pastrami production.
2. To improve the color parameters of TP, the addition of beetroot extract, red pepper extract, or similar natural colorants during the curing or çemen coating stage will strengthen the position of the product in the market by enhancing its visual acceptability. Indeed, the positive Pearson correlation observed between color and appearance in the present study indicates that any improvement in color will be directly reflected in the appearance scores.
3. Infrared drying provided higher panelist acceptability than traditional methods across all sensory parameters of pastrami (flavor, color, appearance, and texture) (44). In the future studies, the use of the infrared drying method in the production of turkey pastrami may further improve the sensory scores, particularly those related to color and texture.
4. It has been reported that vacuum packaging applications suppress lipid oxidation in pastrami produced from turkey meat, thereby preserving color stability and keeping the total bacterial count under control (49). It is anticipated that this strategy may additionally enhance panelist acceptability for TP samples.

Turkey pastrami stood out with the highest protein (38.80 ± 0.08a), the lowest fat (4.40 ± 0.07b), the lowest water activity (0.76 ± 0.01b), and the best chewiness (535.65 N·m) values; however, the fact that its a* value (7.48 ± 0.01c) remained lower compared with beef pastrami restricted the color score given by the panelists (7.00 ± 1.21b). When this finding is evaluated holistically, it is anticipated that turkey pastrami produced with the support of natural colorants and using an appropriate drying method (traditional or infrared) may constitute a healthy, low-cost, and acceptable-by-panelists alternative to beef pastrami.

## Data Availability

The original contributions presented in the study are included in the article/Supplementary material, further inquiries can be directed to the corresponding author.
