# Supplementary material for: Production of pastrami from turkey meat, and based on its physicochemical, microbiological, textural, and sensory characteristics using multivariate statistical analyses
Source: Front Nutr. 2026 Sep 18;13:1943445. doi: 10.3389/fnut.2026.1943445 (PMC13630620; doi:10.3389/fnut.2026.1943445)
Supplement: Supplementary file 1 [file Supplementary_file__1.docx]

|  |  |
| --- | --- |
| (**a**) | (**b**) |
|  |  |
| (**c**) | (**d**) |
|  |  |
| (e) | (f) |
|  |  |
| (g) | (h) |
|  |  |
| (i) | (j) |
|  |  |
| (k) | (l) |

**Supplementary Figure S1.** Stages of TP production using traditional methods: (**a**) Trimming; (**b**) Splitting; (**c**) Salting; (**d**) Washing; (**e**) 1st Drying; (**f**) 1st Pressing; (**g**) 2nd Drying; (**h**) 2nd Pressing; (**i**) 3rd Drying; (**j**) Fenugreek Application; (**k**) 4th Drying; (**l**) Storage.

**Supplementary Table S1.** Correlations between the parameters.

|  | **Appearance** | **Color** | **Odor** | **Flavor** | **Brittleness** | **G. Acceptability** | **Dry Matter** | **pH** | **Salt** | **Ash** | **Aw** | **Protein** | **Fat** |
| --- | --- | --- | --- | --- | --- | --- | --- | --- | --- | --- | --- | --- | --- |
| **Appearance** | **1,000** | 0,769 | 0,505 | 0,537 | 0,657 | 0,700 | -0,012 | -0,405 | 0,315 | -0,037 | -0,152 | 0,307 | -0,427 |
| **Color** | 0,769 | **1,000** | 0,707 | 0,692 | 0,669 | 0,796 | 0,061 | -0,262 | 0,203 | -0,112 | -0,069 | 0,180 | -0,270 |
| **Odor** | 0,505 | 0,707 | **1,000** | 0,815 | 0,750 | 0,841 | 0,046 | -0,238 | 0,158 | -0,174 | -0,008 | 0,130 | -0,257 |
| **Flavor** | 0,537 | 0,692 | 0,815 | **1,000** | 0,724 | 0,832 | 0,098 | -0,083 | 0,079 | -0,113 | -0,037 | 0,064 | -0,067 |
| **Brittleness** | 0,657 | 0,669 | 0,750 | 0,724 | **1,000** | 0,843 | 0,034 | -0,260 | 0,141 | -0,165 | 0,057 | 0,103 | -0,306 |
| **G. Acceptability** | 0,700 | 0,796 | 0,841 | 0,832 | 0,843 | **1,000** | -0,077 | -0,183 | 0,092 | -0,152 | 0,052 | 0,066 | -0,223 |
| **Dry Matter** | -0,012 | 0,061 | 0,046 | 0,098 | 0,034 | -0,077 | **1,000** | 0,117 | -0,131 | 0,041 | 0,146 | -0,161 | 0,154 |
| **pH** | -0,405 | -0,262 | -0,238 | -0,083 | -0,260 | -0,183 | 0,117 | **1,000** | -0,910 | 0,417 | 0,570 | -0,872 | **0,959** |
| **Salt** | 0,315 | 0,203 | 0,158 | 0,079 | 0,141 | 0,092 | -0,131 | -0,910 | **1,000** | -0,417 | -0,849 | **0,990** | -0,762 |
| **Ash** | -0,037 | -0,112 | -0,174 | -0,113 | -0,165 | -0,152 | 0,041 | 0,417 | -0,417 | **1,000** | 0,222 | -0,339 | 0,322 |
| **Aw** | -0,152 | -0,069 | -0,008 | -0,037 | 0,057 | 0,052 | 0,146 | 0,570 | -0,849 | 0,222 | **1,000** | -0,899 | 0,335 |
| **Protein** | 0,307 | 0,180 | 0,130 | 0,064 | 0,103 | 0,066 | -0,161 | -0,872 | **0,990** | -0,339 | -0,899 | **1,000** | -0,712 |
| **Fat** | -0,427 | -0,270 | -0,257 | -0,067 | -0,306 | -0,223 | 0,154 | **0,959** | -0,762 | 0,322 | 0,335 | -0,712 | **1,000** |
| **L* values** | -0,174 | -0,096 | -0,070 | -0,079 | -0,020 | -0,012 | 0,122 | 0,668 | -0,901 | 0,489 | **0,954** | -0,919 | 0,436 |
| **a* values** | -0,251 | -0,173 | -0,219 | -0,026 | -0,318 | -0,246 | 0,049 | 0,364 | 0,048 | 0,104 | -0,549 | 0,131 | 0,602 |
| **b* values** | -0,339 | -0,221 | -0,256 | -0,048 | -0,349 | -0,269 | 0,096 | 0,609 | -0,230 | 0,225 | -0,294 | -0,150 | 0,799 |
| **Hardness** | -0,081 | -0,029 | -0,095 | 0,060 | -0,209 | -0,132 | -0,014 | 0,007 | 0,381 | -0,044 | -0,780 | 0,459 | 0,265 |
| **Adhesiveness** | 0,341 | 0,234 | 0,259 | 0,048 | 0,346 | 0,268 | -0,096 | -0,609 | 0,230 | -0,183 | 0,292 | 0,152 | -0,801 |
| **Springiness** | -0,085 | -0,035 | -0,100 | 0,055 | -0,213 | -0,138 | -0,014 | 0,009 | 0,383 | -0,043 | -0,784 | 0,460 | 0,269 |
| **Cohesiveness** | 0,422 | 0,266 | 0,260 | 0,061 | 0,316 | 0,232 | -0,161 | -0,936 | 0,711 | -0,323 | -0,262 | 0,656 | -0,997 |
| **Gumminess** | 0,373 | 0,239 | 0,194 | 0,087 | 0,183 | 0,139 | -0,173 | -0,954 | **0,977** | -0,359 | -0,774 | **0,970** | -0,849 |
| **Chewiness** | 0,311 | 0,210 | 0,151 | 0,101 | 0,108 | 0,093 | -0,157 | -0,841 | **0,966** | -0,329 | -0,895 | **0,981** | -0,679 |
| **Resilience** | -0,136 | -0,100 | -0,154 | 0,007 | -0,255 | -0,196 | -0,001 | 0,077 | 0,335 | -0,019 | -0,768 | 0,414 | 0,343 |
| **Coliform** | 0,103 | 0,085 | 0,141 | -0,004 | 0,237 | 0,187 | 0,023 | 0,015 | -0,421 | 0,053 | 0,824 | -0,497 | -0,255 |
| **T.Bacteria** | 0,390 | 0,277 | 0,258 | 0,104 | 0,286 | 0,191 | 0,006 | -0,983 | 0,877 | -0,475 | -0,496 | 0,817 | -0,947 |
| **Enterobacter** | -0,142 | -0,069 | -0,009 | -0,042 | 0,058 | 0,054 | 0,125 | 0,562 | -0,848 | 0,244 | **0,999** | -0,893 | 0,321 |
| **Yeast-Mold** | 0,429 | 0,267 | 0,253 | 0,063 | 0,301 | 0,220 | -0,170 | -0,958 | 0,763 | -0,301 | -0,343 | 0,717 | -1,000 |

**Supplementary Table S1.** Correlations between the parameters (continued).

|  | **L* values** | **a* values** | **b* values** | **Hardness** | **Adhesiveness** | **Springiness** | **Cohesiveness** | **Gumminess** | **Chewiness** | **Resilience** | **Coliform** | **Total Bacteria** | **Enterobacter** | **Yeast-Mold** |
| --- | --- | --- | --- | --- | --- | --- | --- | --- | --- | --- | --- | --- | --- | --- |
| **Appearance** | -0,174 | -0,251 | -0,339 | -0,081 | 0,341 | -0,085 | 0,422 | 0,373 | 0,311 | -0,136 | 0,103 | 0,390 | -0,142 | 0,429 |
| **Color** | -0,096 | -0,173 | -0,221 | -0,029 | 0,234 | -0,035 | 0,266 | 0,239 | 0,210 | -0,100 | 0,085 | 0,277 | -0,069 | 0,267 |
| **Odor** | -0,070 | -0,219 | -0,256 | -0,095 | 0,259 | -0,100 | 0,260 | 0,194 | 0,151 | -0,154 | 0,141 | 0,258 | -0,009 | 0,253 |
| **Flavor** | -0,079 | -0,026 | -0,048 | 0,060 | 0,048 | 0,055 | 0,061 | 0,087 | 0,101 | 0,007 | -0,004 | 0,104 | -0,042 | 0,063 |
| **Brittleness** | -0,020 | -0,318 | -0,349 | -0,209 | 0,346 | -0,213 | 0,316 | 0,183 | 0,108 | -0,255 | 0,237 | 0,286 | 0,058 | 0,301 |
| **G. Acceptability** | -0,012 | -0,246 | -0,269 | -0,132 | 0,268 | -0,138 | 0,232 | 0,139 | 0,093 | -0,196 | 0,187 | 0,191 | 0,054 | 0,220 |
| **Dry Matter** | 0,122 | 0,049 | 0,096 | -0,014 | -0,096 | -0,014 | -0,161 | -0,173 | -0,157 | -0,001 | 0,023 | 0,006 | 0,125 | -0,170 |
| **pH** | 0,668 | 0,364 | 0,609 | 0,007 | -0,609 | 0,009 | -0,936 | -0,954 | -0,841 | 0,077 | 0,015 | -0,983 | 0,562 | -0,958 |
| **Salt** | -0,901 | 0,048 | -0,230 | 0,381 | 0,230 | 0,383 | 0,711 | **0,977** | **0,966** | 0,335 | -0,421 | 0,877 | -0,848 | 0,763 |
| **Ash** | 0,489 | 0,104 | 0,225 | -0,044 | -0,183 | -0,043 | -0,323 | -0,359 | -0,329 | -0,019 | 0,053 | -0,475 | 0,244 | -0,301 |
| **Aw** | **0,954** | -0,549 | -0,294 | -0,780 | 0,292 | -0,784 | -0,262 | -0,774 | -0,895 | -0,768 | 0,824 | -0,496 | **0,999** | -0,343 |
| **Protein** | -0,919 | 0,131 | -0,150 | 0,459 | 0,152 | 0,460 | 0,656 | **0,970** | **0,981** | 0,414 | -0,497 | 0,817 | -0,893 | 0,717 |
| **Fat** | 0,436 | 0,602 | 0,799 | 0,265 | -0,801 | 0,269 | -0,997 | -0,849 | -0,679 | 0,343 | -0,255 | -0,947 | 0,321 | -1,000 |
| **L* values** | **1,000** | -0,419 | -0,150 | -0,681 | 0,162 | -0,684 | -0,372 | -0,822 | -0,911 | -0,659 | 0,723 | -0,622 | **0,959** | -0,437 |
| **a* values** | -0,419 | **1,000** | **0,959** | 0,895 | -0,959 | 0,902 | -0,661 | -0,097 | 0,156 | **0,955** | -0,925 | -0,409 | -0,562 | -0,595 |
| **b* values** | -0,150 | **0,959** | **1,000** | 0,761 | -0,997 | 0,768 | -0,843 | -0,370 | -0,121 | 0,834 | -0,781 | -0,638 | -0,309 | -0,793 |
| **Hardness** | -0,681 | 0,895 | 0,761 | **1,000** | -0,751 | **1,000** | -0,340 | 0,280 | 0,527 | **0,968** | -0,959 | -0,069 | -0,788 | -0,258 |
| **Adhesiveness** | 0,162 | -0,959 | -0,997 | -0,751 | **1,000** | -0,759 | 0,844 | 0,375 | 0,130 | -0,833 | 0,781 | 0,636 | 0,308 | 0,797 |
| **Springiness** | -0,684 | 0,902 | 0,768 | **1,000** | -0,759 | **1,000** | -0,344 | 0,277 | 0,524 | **0,974** | -0,966 | -0,071 | -0,792 | -0,262 |
| **Cohesiveness** | -0,372 | -0,661 | -0,843 | -0,340 | 0,844 | -0,344 | **1,000** | 0,806 | 0,619 | -0,415 | 0,328 | 0,928 | -0,248 | **0,996** |
| **Gumminess** | -0,822 | -0,097 | -0,370 | 0,280 | 0,375 | 0,277 | 0,806 | **1,000** | **0,963** | 0,199 | -0,285 | 0,908 | -0,764 | 0,853 |
| **Chewiness** | -0,911 | 0,156 | -0,121 | 0,527 | 0,130 | 0,524 | 0,619 | **0,963** | **1,000** | 0,439 | -0,511 | 0,783 | -0,889 | 0,684 |
| **Resilience** | -0,659 | **0,955** | 0,834 | **0,968** | -0,833 | **0,974** | -0,415 | 0,199 | 0,439 | **1,000** | -0,995 | -0,135 | -0,778 | -0,336 |
| **Coliform** | 0,723 | -0,925 | -0,781 | -0,959 | 0,781 | -0,966 | 0,328 | -0,285 | -0,511 | -0,995 | **1,000** | 0,046 | 0,834 | 0,248 |
| **T.Bacteria** | -0,622 | -0,409 | -0,638 | -0,069 | 0,636 | -0,071 | 0,928 | 0,908 | 0,783 | -0,135 | 0,046 | **1,000** | -0,494 | 0,941 |
| **Enterobacter** | **0,959** | -0,562 | -0,309 | -0,788 | 0,308 | -0,792 | -0,248 | -0,764 | -0,889 | -0,778 | 0,834 | -0,494 | **1,000** | -0,328 |
| **Yeast-Mold** | -0,437 | -0,595 | -0,793 | -0,258 | 0,797 | -0,262 | **0,996** | 0,853 | 0,684 | -0,336 | 0,248 | 0,941 | -0,328 | **1,000** |
